# Re-Irradiation Using Brachytherapy for Recurrent Intracranial Tumors: A Systematic Review and Meta-Analysis of the Literature

**DOI:** 10.7759/cureus.9666

**Published:** 2020-08-11

**Authors:** Mehee Choi, Joseph M Zabramski

**Affiliations:** 1 Radiation Oncology, GT Medical Technologies, Inc., Tempe, USA; 2 Neurosurgery, Barrow Neurological Institute, Phoenix, USA

**Keywords:** brachytherapy, re-irradiation, recurrence, brain tumor, brain metastasis, meningioma, high-grade glioma, surgically-targeted radiation therapy

## Abstract

Introduction

We aim to compare the efficacy and toxicity of re-irradiation using brachytherapy for patients with locally recurrent brain tumors after previous radiation therapy.

Methods

We performed a systematic review of the major biomedical databases from 2005 to 2020 for eligible studies where patients were treated with re-irradiation for recurrent same site tumors using brachytherapy. Tumor types included high-grade gliomas (HGG) (World Health Organization (WHO) Grades 3 and 4), meningiomas, and metastases. The outcomes of interest were median overall survival (OS) and progression-free survival (PFS) after re-irradiation, the incidence of radiation necrosis (RN), and other relevant radiation-related adverse events (AE). We used a fixed-effect meta-analysis regression moderation model to compared results of interstitial versus intracavitary therapy, treatment with low-dose-rate (LDR) versus high-dose-rate (HDR) techniques, and outcomes by tumor type.

Results

The search resulted in a total of 194 articles. A total of 16 articles with 695 patients fulfilled the inclusion criteria and were selected for analysis. For high-grade glioma, meningioma, and brain metastasis the pooled meta-analysis showed mean symptomatic RN rates of 3.3% (standard error (SE) = 0.8%), 17.3% (SE = 5.0%), and 22.4% (SE = 7.0%), respectively, and mean rates of RN requiring surgical intervention of 3.0% (SE = 1.0%), 11.9% (SE = 5.3%), and 10.0% (SE = 7.3%), respectively.

The mean symptomatic RN rates in the meta-analysis comparing interstitial versus intracavitary therapy were 3.4% and 4.9%, respectively (p = 0.36), and for the comparison of LDR versus HDR, the rates were 2.6% and 5.7%, respectively (p = 0.046). In comparing the symptomatic RN rates in comparison to HGG versus meningioma, the means were 3.3% and 17.3%, respectively (p = 0.006), and in HGG versus metastatic tumors, the means were 3.3% and 22.4%, respectively (p = 0.007). There was no significant difference in rates of RN requiring surgery in any of these groups. Due to the small number of studies and inconsistent recording of OS and PFS, statistical analysis of these parameters could not be performed.

Conclusion

Published literature on the same site re-irradiation using brachytherapy for recurrent brain tumors is highly limited, with inconsistent reporting of safety and efficacy outcomes. To overcome these shortcomings, we utilized a structured meta-analysis approach to show that re-irradiation with modern brachytherapy is generally safe in terms of the risks of symptomatic RN. We also found that symptomatic RN rates for brachytherapy are significantly lower in recurrent HGG compared to recurrent meningiomas (p = 0.006) and metastatic tumors (p = 0.007). Re-irradiation with brachytherapy is a feasible option for appropriately selected patients. The availability of Cesium-131 (Cs-131) shows promise in reducing toxicity while achieving excellent local control due to its physical properties, and the recent introduction of a novel surgically targeted radiation therapy device, that makes brachytherapy less technically demanding, may allow for more widespread adoption. Prospective trials with consistent reporting of endpoints are needed to explore whether these advances improve safety and efficacy in patients with recurrent, previously irradiated tumors.

## Introduction

An estimated 87,240 new cases of primary brain and other central nervous system (CNS) tumors are expected to be diagnosed in the United States in 2020 [1]. Glioblastoma, the most commonly occurring primary malignant brain tumor, and meningioma, the most common non-malignant tumor, represent 30% and 34.7% of cases, respectively. Brain metastases account for an even larger number of intracranial lesions and occur up to 10 times more frequently than primary brain tumors, with as many as 8% - 10% of patients with cancer being affected by symptomatic metastatic brain tumors and the incidence rising because of better control of the systemic disease [2]. The mainstay of therapy for primary tumors and large metastatic tumors is maximum safe resection followed by adjuvant therapy, which depending on the pathology, commonly includes radiotherapy to the tumor bed, +/- chemotherapy [2]. Despite this multimodality approach, recurrence rates are high.

Effective management of local tumor recurrence in patients who have undergone previous irradiation is problematic. While re-irradiation can potentially prolong survival in patients with recurrent brain tumors, its use has been limited due to concerns of increased risks of toxicity to surrounding normal brain, particularly when the recurrent lesion lies within a previous field of treatment [3-4]. Re-irradiation using brachytherapy may provide a safer alternative in such cases [3, 5-7].

Brachytherapy was first used for intracranial tumors as early as 1923 by Harvey Cushing who implanted two tubes of radium for 28 hours in the surgical cavity of a 45-year-old man following resection of malignant glioma [8]. The patient required a second operation for an abscess but did relatively well, surviving for 63 months before succumbing to the recurrent tumor. A total of 10 additional cases followed between 1928 and 1931 using so-called “radium bombs” composed of radium needles in a rubber sponge-wrapped in rubber tissue, the size of the implant corresponding approximately to the size of the cavity left by the malignant tumor. Since that time, Iodine-125 (I-125) has been the most frequently used isotope for the brachytherapy of brain tumors. Other modern brachytherapy isotopes include phosphorus-32 (P-32), iridium-192 (Ir-192), and more recently, cesium-131 (Cs-131) [7, 9-10].

Modern brachytherapy for brain tumors can be essentially divided into two categories: interstitial and intracavitary techniques [9-10]. Both techniques require close cooperation between the neurosurgery and radiation oncology teams. With interstitial therapy, radiation sources are inserted directly into the tumor using stereotactic techniques and left permanently in place or removed after the prescribed dose has been delivered [9]. With intracavitary brachytherapy, the patient undergoes craniotomy with maximum safe resection of the tumor followed by placement of a radiation source(s) directly along the walls of the tumor cavity [9]. The intracavitary placement of permanent sources at the time of resection has the added benefit of initiating radiation therapy immediately and at a time when tumor burden has been surgically minimized.

Despite its potential advantages, brachytherapy is rarely used in the management of recurrent brain tumors largely due to the technical demands of treatment, and the high rates of radiation necrosis reported with the traditional isotope, I-125. More recently, a novel surgically targeted radiation therapy (STaRT) brachytherapy device has become clinically available that minimizes the technical issues associated with intracavitary brachytherapy [6]. This device consists of Cs-131 seeds positioned 1 cm apart within a collagen carrier tile that is permanently implanted at the time of surgery, typically taking less than 5 minutes to place. Cs-131 may have some advantage as a brachytherapy source. While both I-125 and Cs-131 are low-energy gamma emitters (30 keV), Cs-131 has a shorter half-life than I-125 (9.7 days versus 59.4 days, respectively) [6, 10]. The ability of Cs-131 sources to deliver 50% of the treatment dose in 10 days makes this isotope a better choice for rapidly growing tumors [11].

Given the recent developments in brain brachytherapy, we sought to compare the clinical efficacy and toxicity outcomes through a meta-analysis of the literature on the treatment of locally recurrent brain tumors with the same site re-irradiation using various forms of brachytherapy.

## Materials and methods

Search strategy and selection criteria for studies

**Figure 1:**
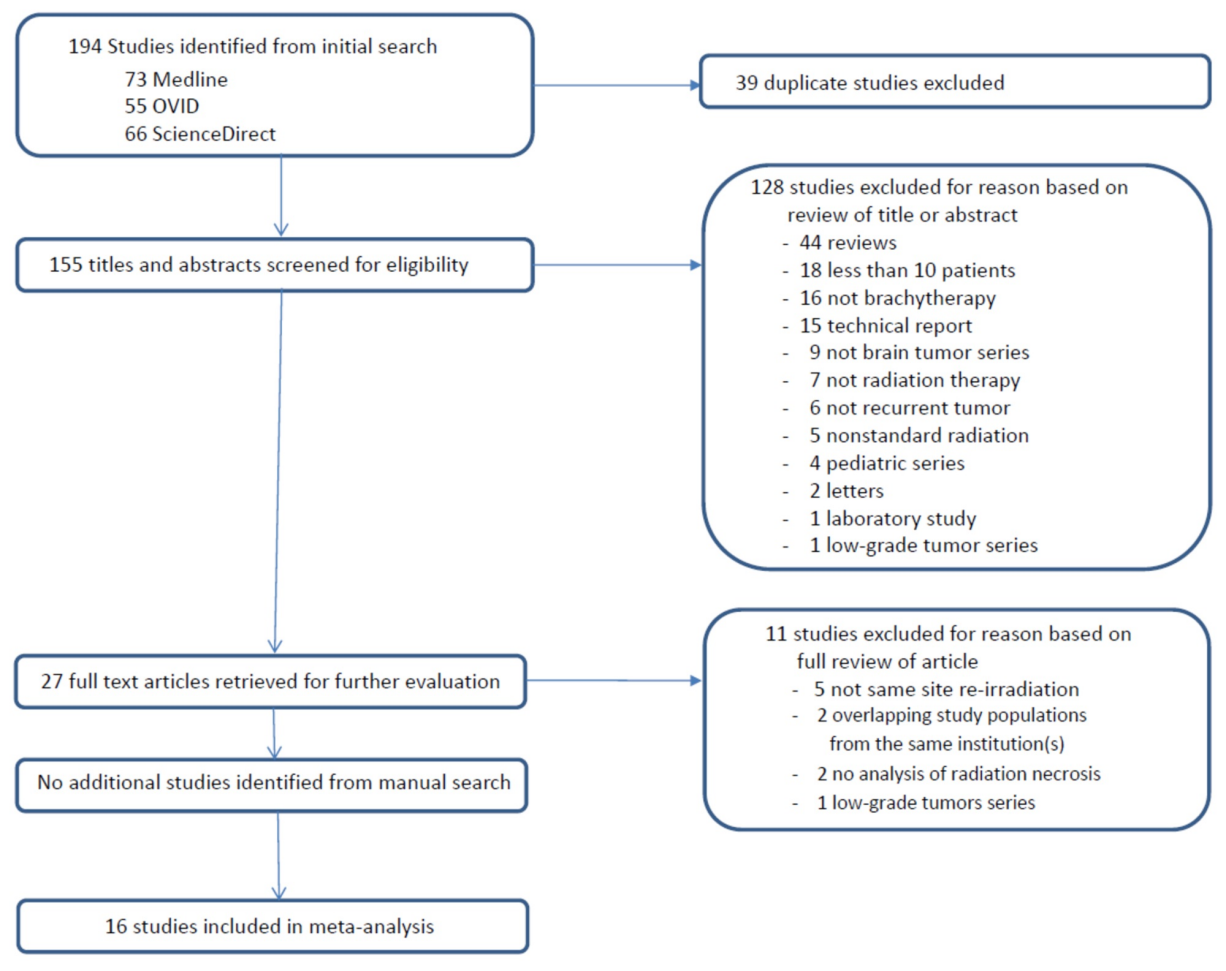
PRISMA (Preferred Reporting Items for Systematic Reviews and Meta-analyses) flow diagram for the selection of studies

We conducted a search of the following electronic databases: MEDLINE (via PubMed), OVID (via OpenAthens), and ScienceDirect. Terms used in the searches were “brachytherapy,” “glioblastoma,” “high-grade glioma,” brain metastases,” “cerebral metastases,” “meningioma,” and “recurrent”. We limited the search to studies published from January 2005 to April 2020 in the English language. We also searched the reference lists of identified studies to find relevant articles. The PRISMA (Preferred Reporting Items for Systematic Reviews and Meta-analyses) flow diagram is shown in Figure 1.

Two authors independently screened the titles and abstracts. Studies that met the following criteria were included: (1) patients received a primary course of radiotherapy for the initial diagnosis, (2) histologically and/or radiologically-proven locally recurrent tumors, (3) same site re-irradiation using brachytherapy, (4) at least a six-month follow-up, and (5) report of the incidence of radiation necrosis (RN), as well as other relevant radiation-related complications [12]. Additional outcomes of interest included median overall survival (OS) and median PFS after re-irradiation. We excluded review articles, pediatric studies, technical reports, case series with less than 10 patients, as well as letters and laboratory reports.

Data collection and extraction

Two reviewers (MC, JZ) evaluated the titles and abstracts of the search results independently. The full texts of articles that met the inclusion criteria were retrieved for further evaluation. Discrepancies in article selection were resolved by consensus after detailed discussions. The same two reviewers then extracted the data independently from the full-text articles using standardized data collection forms. Data that were collected included publication details, study methodology, sample size, pathologic tumor diagnosis, type of primary radiation intervention and dose, type of brachytherapy treatment and dose, the interval between primary radiation and re-irradiation treatment, and clinical outcomes, including median OS from diagnosis, median OS following re-irradiation, median PFS, rates of RN, and other neurological complications related to the method of brachytherapy. Studies in our systematic review and meta-analysis diagnosed RN by magnetic resonance imaging, positron emission tomography, or histological confirmation. Other neurological complications included acute radiation effects, intracerebral hemorrhage, cerebral spinal fluid leaks, infection, seizures, and treatment-related wound complications. Once again, any discrepancies in the collected data were resolved by consensus.

Brachytherapy studies were grouped by tumor type, interstitial or intracavitary therapy, and the type of source (low-dose-rate (LDR) brachytherapy using I-125 or Cs-131 or high-dose-rate (HDR) brachytherapy with I-192). 

Data quality assessment

We assessed the quality of each study using a simplified version of the Oxford Centre for Evidence-Based Medicine (OCEBM) as presented in Table 1.

**Table 1:** Levels of Evidence (Adapted From the Oxford Centre for Evidence-Based Medicine) RCT: randomized controlled trial

Level of Evidence	Study Design	Randomization	Control Group
Level 1	High-quality RCT with statistically significant differences, or no statistically significant difference but narrow confidence intervals	Yes	Yes
Level 2	Lesser quality RCT (e.g., < 80% follow-up, no blinding, improper randomization	Yes	Yes
Level 2	Prospective comparative study	Yes	Yes
Level 3	Retrospective cohort study	No	Yes
Level 3	Case-control study	No	Yes
Level 4	Prospective case series	No	No
Level 4	Retrospective case series	No	No
Level 5	Case Report	No	No
Level 5	Expert Opinion	No	No

Statistical methods

The goal of this study was to examine the difference in univariate statistics (typical rates and duration) produced by different studies that are grouped by different treatment approaches. These models rely on weighting the means by the inverse of the associated sampling variance (σ, defined below).

This set of descriptive comparisons of study outcomes was achieved using a fixed-effect (FE) meta-analysis regression moderation model [13]. This model is defined as follows: for a set of i={1,2,…,N} studies in which each study is a member of one of the j={1,2,…,J} groups, the model estimates

$\mu_i = \beta_0 + \sum_\mathcal{j}^{\mathcal{J}-1}{\beta_\mathcal{j}d(i\in \mathcal{j})}$

where the slopes (βs) represent the differences in the outcome statistic between the identified group of studies and the reference group of studies (β0 is the average outcome for the reference group); d() is an indicator function identifying a study’s group membership. In addition, the residual weighted sum of squares heterogeneity statistic is estimated with the Q, defined as

$Q = \frac{\sum_i{(\mu_i - (\beta_0 + \sum_\mathcal{j}^{\mathcal{J}-1}{\beta_\mathcal{j}d(i\in \mathcal{j}))^2}}}{\sigma_i}$

The value of I2 is an approximate measure of the extent to which the observed variation across studies is due to resulting heterogeneity (rather than chance), defined as

$I^2 = 100\times \frac{Q-df}{Q}$

where df=N−J (the number of studies minus the number of groups). The value of I2 is truncated to 0 when Q−df is negative.

Outcome Types 

Meta-analysis is focused on summarizing study means (μ) weighted by the inverse of the means’ sampling variances (or inverse of the standard-error squared). The standard error is noted as σ. For many studies, we computed standard errors based on available data reported. Medians by nature do not have a straightforward sampling variance formula as they are a quantity based on the empirical distribution of the sample and thus were converted to means using methods proposed by Hozo et al. [14].

The rates reported in the available studies were used without alteration. The standard error of a rate or proportion is well known as simply a function of the rate (r) and reported sample size n:

$\sigma = \sqrt{\frac{r(1-r)}{n}}$

Medians are statistics that typically do not allow straightforward estimates for sampling variances, as they are a description of a value at the center of the empirical cumulative distribution.

For the meta-analysis, we implemented a typical procedure for meta-analysis of medians by converting the medians into means and estimating the sampling variance of the means using the median m, the sample size, n, and the range comprised of the minimum a and maximum b [14].

The mean is estimated by expression (4)

$\mu = \frac{a+2m+b}{4} + \frac{a-2m+b}{4n}$

and the standard error of the mean is estimated by the square-root of expression (15) in the study by Hozo et al. [14]:

$\sigma = \sqrt{\frac{n+1}{48(n-1)^2}((n^2+3)(a-2m+b)^2+4n^2(b-a)^2)}$

Procedure

We estimated the fixed effects moderator models using the metafor package written for the R statistical environment (R Foundation for Statistical Computing, Vienna, Austria), specifically, the procedure implemented by the rma.uni function, noting the fixed effect argument method = "FE" and setting the confidence level to 90% (α = .1, two-tailed tests) [15].

## Results

We identified 194 studies in the initial search, and 39 were excluded as duplicates. An additional 128 studies were excluded as they did not fulfill the inclusion criteria at the initial screening of their titles and abstracts. The full text of the remaining 27 studies was reviewed, and 11 of these were excluded as they did not meet the pre-established inclusion criteria, the most common reason being 'Not same site re-irradiation' (Figure 1). The remaining 16 eligible studies included a total of 695 patients (Table 2) [3, 5, 16-29]. Of these, 12 studies used low-dose-rate (LDR) brachytherapy with I-125 or Cs-131, and four studies used high-dose-rate (HDR) brachytherapy with Ir-192 for re-irradiation. Nine studies reported the results for the treatment of recurrent high-grade gliomas (World Health Organization (WHO) Grade III and IV), four studies reporting recurrent meningioma, and three studies reporting recurrent metastatic tumors.

**Table 2:** Included Studies BRT: brachytherapy; Cs-131: Cesium-131; Gd - grade using the Common Terminology Criteria for Adverse Events (CTCAE, version 5.0); HGG: high-grade glioma; I-125: Iodine-125; Ir-192: Iridium-192; LRT-BRT: time from first last radiation therapy to brachytherapy; Mening: meningioma; Mets: metastases; NR: not reported; OS: overall survival; PCC: prospective case-control; PCS: prospective case series; RCC: retrospective case-control; RCS: retrospective case series; RN: radiation necrosis; RT: radiation therapy; TB: tumor border; Tx: treatment

	Study	N	Study Type	Tumor Type	Type of Implant	Source	Median Dose (Gy)	Depth (mm)	LRT-BRT (mo.)	RN % (Gd 3)	RN % (Gd 4)	OS BRT (mo)	Study Quality
1	Chan et al. [16]	24	PCS	HGG	Intracavitary	I-125 GliaSite	53*	5	NR	8.3	8.3	9.1	4
2	Gabayan et al. [17]	95	RCS	HGG	Intracavitary	I-125 GliaSite	60	10	12.6	2.1	2.1	9.1	4
3	Tselis et al. [29]	84	RCS	HGG	Interstitial	Ir-192	40	TB	NR	2.4	0	9.2	4
4	Darakchiev et al. [5]	34	PCS	HGG	Intracavitary	I-125	120	5	NR	23.5	11.8	17.2	4
5	Fabrini et al. [28]	21	RCS	HGG	Intracavitary	Ir-192	18	5	8.6	0	0	5.5	4
6	Gobitti et al. [18]	15	RCS	HGG	Intracavitary	I-125 GliaSite	45	10	28	20	20	13	4
7	Archavlis et al. [26]	46	PCC	HGG	Interstitial	Ir-192	40	TB	NR	8.7	0	8	3
8	Schwartz et al. [25]	68	RCS	HGG	Interstitial	1-125	50	TB	NR	8.8	0	13.4	4
9	Chatzikonstantinou et al. [27]	135	RCS	HGG	Interstitial	Ir-192	40	TB	9.3	2.2	2.2	9.2	4
10	Magill et al. [21]	42	RCS	Mening	Intracavitary	I-125	120	5	NR	19	11.9	39.6	4
11	Brachman et al. [3]	19	PCS	Mening	Intracavitary	Cs-131	63	5	NR	10.5	0	26	4
12	Koch et al. [20]	15	RCS	Mening	Intracavitary	I-125 / Cs-131	100	5	14	40	0	12.5	4
13	Mooney et al. [22]	11	RCC	Mening	Intracavitary	I-125 / Cs-131	100	5	NR	27.3	0	NR	3
14	Huang et al. [19]	21	RCS	Mets	Intracavitary	I-125	300	5	11.1	19	10	7.3	4
15	Ruge et al. [24]	27	RCS	Mets	Interstitial	I-125	50	TB	9	0	0	14.8	4
16	Raleigh et al. [23]	38	RCS	Mets	Intracavitary	I-125	263	5	NR	25	0	12	4

The quality of the summarized evidence is listed in Table 1. In all, there were 14 Level 4 studies, including 11 retrospective case reviews and three prospective case reviews, and two Level 3 case-control studies [3, 5, 16-29].

Meta-analysis was carried out to obtain pooled means for the rates of symptomatic RN and RN requiring surgery for brachytherapy in recurrent HGG, meningiomas, and metastases. Additional meta-analysis evaluations were performed comparing the rates of symptomatic RN and RN requiring surgery for interstitial versus intracavitary therapy, for HDR versus LDR sources, and to compare the rates for treatment of recurrent HGG versus meningioma and metastases. Because of inconsistent reporting of OS and PFS, meta-analysis evaluations could not be performed for these outcomes.

High-grade glioma (WHO grades III & IV) studies

Of the nine recurrent high-grade glioma studies, three utilized interstitial HDR, one utilized interstitial LDR, one utilized intracavitary HDR, and four utilized intracavitary LDR techniques [5, 16-18, 25-29]. Study characteristics are presented in Table 2 (Entries 1 - 9). The median dose in this group of studies was 45 Gy (range: 18 - 120 Gy) prescribed to the tumor surface for interstitial techniques and at a depth of 5 mm or 10 mm for intracavitary techniques. Two studies used I-125 seeds, three used GliaSite I-125 (Cytyc Surgical Products, Palo Alto, CA), and four studies used Ir-192 [5, 16-18, 25-29]. Three studies included concurrent chemotherapy with temozolomide, fotemustine, or carmustine wafers [5, 26, 28].

The pooled meta-analysis showed a mean symptomatic RN rate of 3.3%. (SE: 0.8), and the mean rate of RN requiring the surgical intervention of 3.0% (SE: 1.0). Other serious adverse events (AEs) are listed in Table 3 (Entries 1 - 9), including wound healing complications reported in six of nine studies (mean: 3.9%, range: 0 to 12%), cerebral spinal fluid (CSF) leak in three studies (mean: 1.9%, range: 0 to 9.5%), intracerebral hemorrhage (ICH) related to interstitial catheter placement in three studies (mean: 1.5%, range: 0 to 6%), meningitis, seizures, and subgaleal fluid collections (hygromas) in two studies each, and wound infection, stroke, and hydrocephalus each reported in one study each. 

**Table 3:** Other Serious Adverse Events - All Events Are Reported as Percentages (%) Cs-131: Cesium-131; CSF: cerebral spinal fluid; CVA: cerebrovascular accident; HGG: high-grade glioma; HCP: hydrocephalus; ICH: intracerebral hemorrhage; I-125: Iodine-125; Ir-192: Iridium-192; Mening: meningioma; Mets: metastases; NPH: normal pressure hydrocephalus

	Study	Tumor Type	Type of Implant	Source	Wound Healing	CSF Leak	Meningitis	Wound Infection	ICH	Seizures	Poor Seed placement	CVA	NPH/HCP	Hygroma
1	Chan et al. [16]	HGG	Intracavitary	I-125 GliaSite	4.2	–	–	–	–	–	–	–	–	–
2	Gabayan et al. [17]	HGG	Intracavitary	I-125 GliaSite	2	3	–	2	–	2	–	1	1	1
3	Tselis et al. [29]	HGG	Interstitial	Ir-192	–	–	1.2	–	2.4	–	–	–	–	–
4	Darakchiev et al. [5]	HGG	Intracavitary	I-125	12	–	–	–	–	–	–	–	–	–
5	Fabrini et al. [28]	HGG	Intracavitary	Ir-192	9.5	9.5	–	–	5	–	–	–	–	–
6	Gobitti et al. [18]	HGG	Intracavitary	I-125 GliaSite	–	–	–	–	–	–	–	–	–	6.7
7	Archavlis et al. [26]	HGG	Interstitial	Ir-192	4.3	4.3	4.3	–	–	–	–	–	–	–
8	Schwartz et al. [25]	HGG	Interstitial	1-125	2.9	–	–	–	–	–	2.9	–	–	–
9	Chatzikonstantinou et al. [27]	HGG	Interstitial	Ir-192	–	–	–	–	6	0.7	–	–	–	–
10	Magill et al. [21]	Mening	Intracavitary	I-125	14	–	–	7	–	–	–	–	8	5
11	Brachman et al. [3]	Mening	Intracavitary	Cs-131	10	–	–	–	–	5	–	–	–	5
12	Koch et al. [20]	Mening	Intracavitary	I-125/Cs-131	40	–	–	–	–	–	–	–	–	–
13	Mooney et al. [22]	Mening	Intracavitary	I-125/Cs-131	9	–	–	–	–	27	–	–	–	9
14	Huang et al. [19]	Mets	Intracavitary	I-125	–	–	–	–	–	–	–	–	–	13
15	Ruge et al. [24]	Mets	Interstitial	I-125	3.7	–	–	–	–	–	–	–	–	–
16	Raleigh et al. [23]	Mets	Intracavitary	I-125	6	–	–	–	–	–	–	–	–	–

Meningioma studies

Of the four recurrent meningioma studies, all utilized intracavitary LDR techniques with I-125 or Cs-131 [3, 20-22]. Study characteristics are presented in Table 2 (Entries 10 - 13). The median dose in these studies was 100 Gy (range: 63 to 120 Gy) at a depth of 5 mm. Three studies used I-125 or Cs-131 seeds imbedded in the absorbable suture, and one study utilized a novel collagen tile carrier for seed placement [3, 20-22].

The pooled meta-analysis showed a mean symptomatic RN rate of 17.3% (SE: 5.0), and the mean rate of RN requiring the surgical intervention of 11.9% (SE: 5.3). Other serious AEs (Table 3, Entries 10 - 13) included wound healing complications in all four studies (mean: 18.2%, range: 9 to 40%), hygromas in three studies (mean: 4.7%, range: 0 to 9%), seizures in two studies (mean: 8%, range: 0 to 27%), and wound infection and hydrocephalus in one study each. 

Brain metastasis studies

Of the three recurrent metastasis studies, two utilized intracavitary LDR brachytherapy in combination with surgery and one utilized interstitial LDR brachytherapy without resection [19, 23-24]. Study characteristics are presented in Table 2 (Entries 14 - 16). The median dose was 263 Gy (range: 50 to 300 Gy) prescribed at a depth for 5 mm for intracavitary treatments and at the tumor surface for interstitial treatments. All three studies used I-125. Intracavitary treatment was achieved via individual seed placement [19, 23]. For interstitial therapy, catheters were placed under stereotactic guidance, loaded with I-125 seeds, and removed after 42 days under local anesthesia [24].

The pooled meta-analysis showed a mean symptomatic RN rate of 22.4% (SE: 7.0), and the mean rate of RN requiring the surgical intervention of 10.0% (SE: 7.3). Other serious AEs were wound complications reported in one study (mean: 3.2%, range: 0 to 6%), wound infection requiring surgery, and leptomeningeal spread of tumor were reported in one study each (Table 3, Entries 14 - 16).

Comparison of interstitial versus intracavitary therapy 

There were five studies reporting results for interstitial therapy, and 11 intracavitary studies available for evaluation [3, 5, 16-29]. The median dose for interstitial studies was 9.2 Gy (range: 8 to 14.8 Gy) prescribed to the tumor surface. Four of the studies used Ir-192 and one used I-125 [24, 26-29]. The median dose for intracavitary therapy was 81 Gy (range: 18 to 300 Gy) at a depth of 5 mm or 10 mm. Six of the studies utilized I-125 sources, two reported the use of both I-125 and Cs-131, and one used only Cs-131 [3, 5, 17-23].

**Figure 2:**
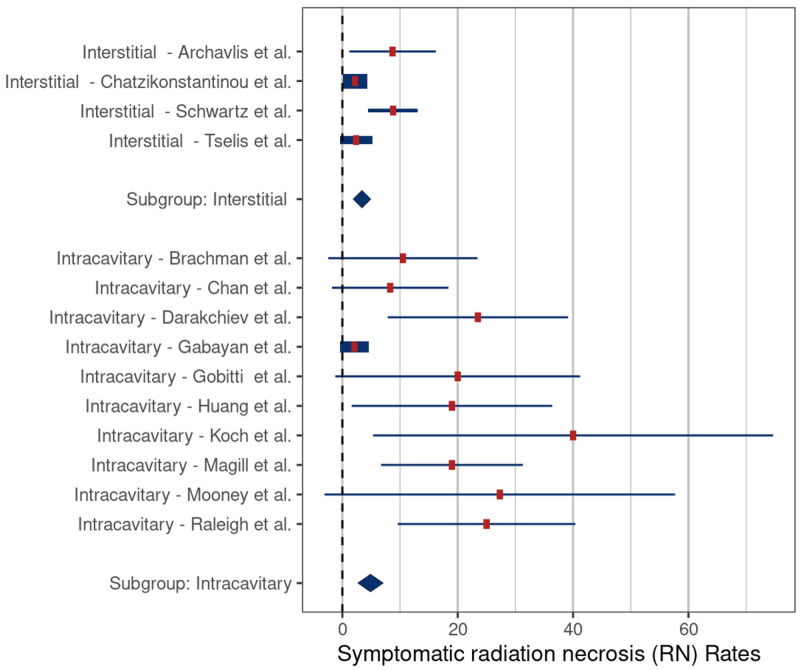
Comparison of rates of symptomatic radiation necrosis – interstitial versus intracavitary brachytherapy (p = 0.36) Comparison based on included studies [3, 5, 16-23, 25-27, 29].

**Figure 3:**
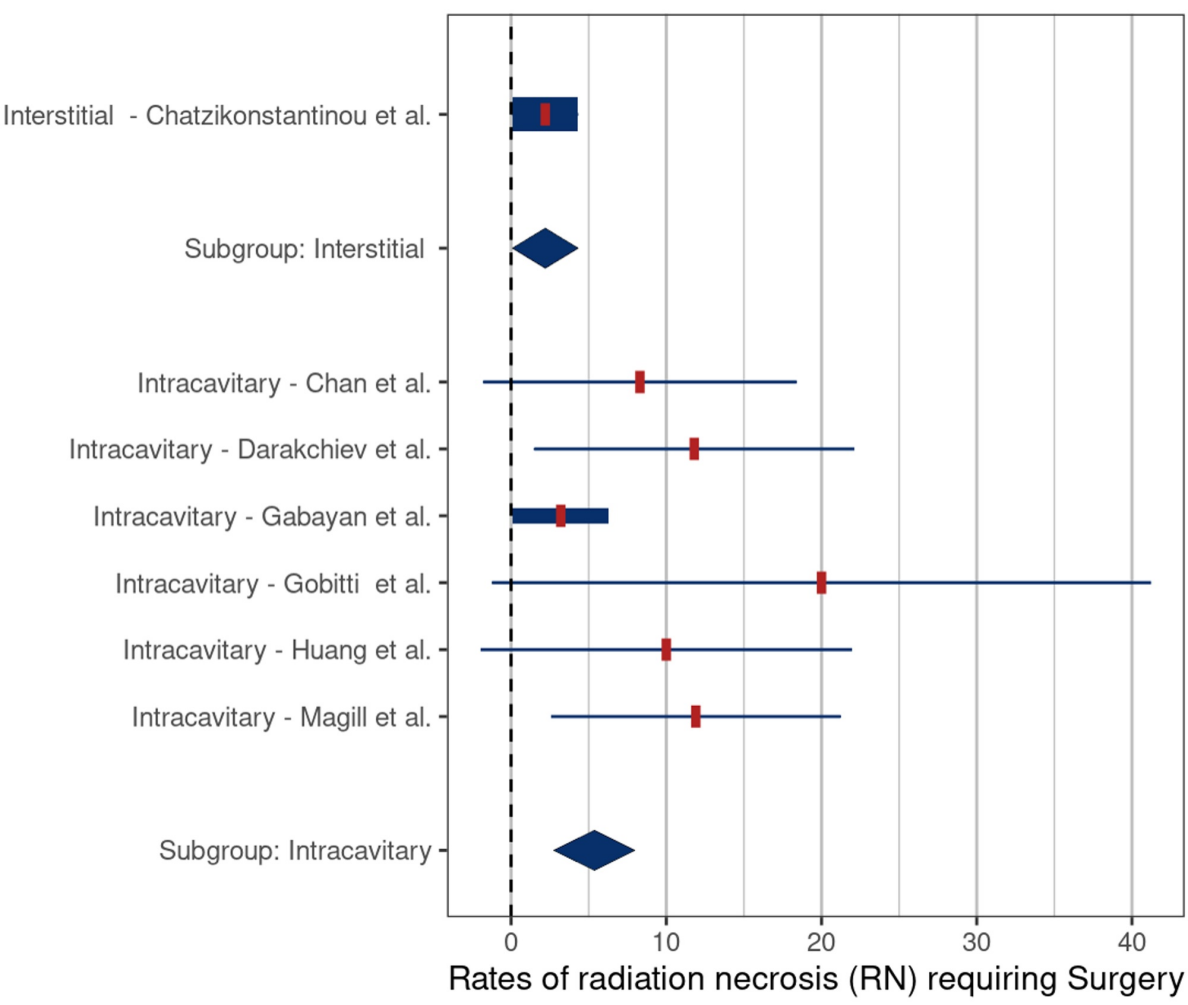
Comparison of rates of radiation necrosis requiring surgery – interstitial versus intracavitary brachytherapy (p = 0.12) Comparison based on included studies [5, 16-19, 21, 27].

The mean rates of symptomatic RN and RN requiring surgery were 3.4% (SE: 0.9) and 4.9% (SE: 1.6) (p = 0.36) (Figure 2) and 2.2% (SE: 1.3) and 5.4% (SE: 2.0) (p = 0.12) for interstitial versus intracavitary therapy, respectively (Figure 3).

Comparison of HDR versus LDR techniques

There were four HDR studies and 12 LDR studies for evaluation [3, 5, 16-29]. The median dose for the HDR studies was 40 Gy (range: 18 to 40 Gy) prescribed to the tumor surface. All HDR studies used Ir-192 sources. The median dose for the LDR studies was 81 Gy (range: 45 to 300 Gy) at a depth of 5 mm or 10 mm. Nine of the LDR studies utilized I-125 sources, two reported the use of both I-125 and Cs-131, and one used only Cs-131 [3, 5, 16-25].

**Figure 4:**
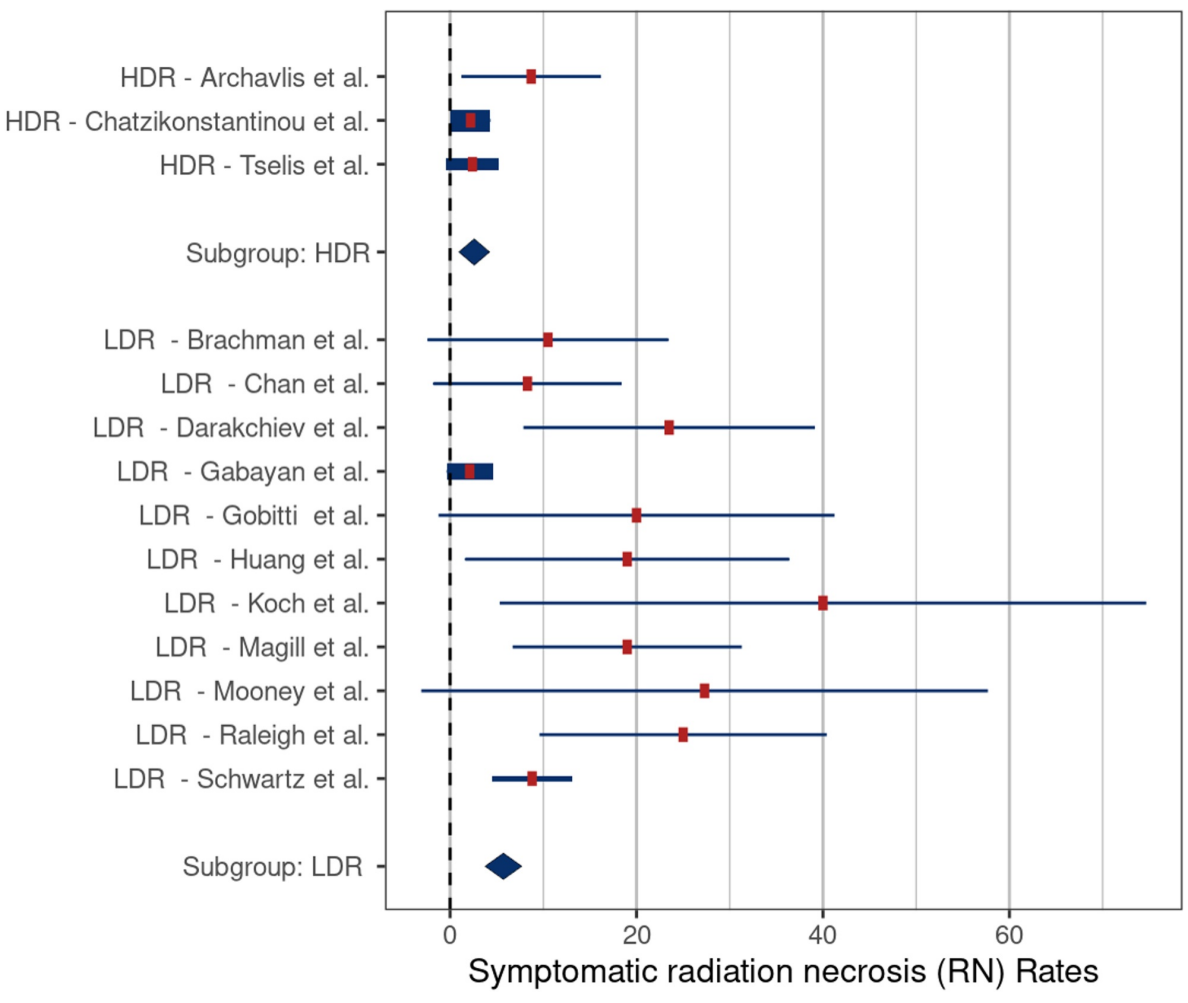
Comparison of rates of symptomatic radiation necrosis – high-dose-rate (HDR) versus low-dose-rate (LDR) brachytherapy (p = 0.046) Comparison based on included studies [3, 5, 16-23, 25-27, 29]

**Figure 5:**
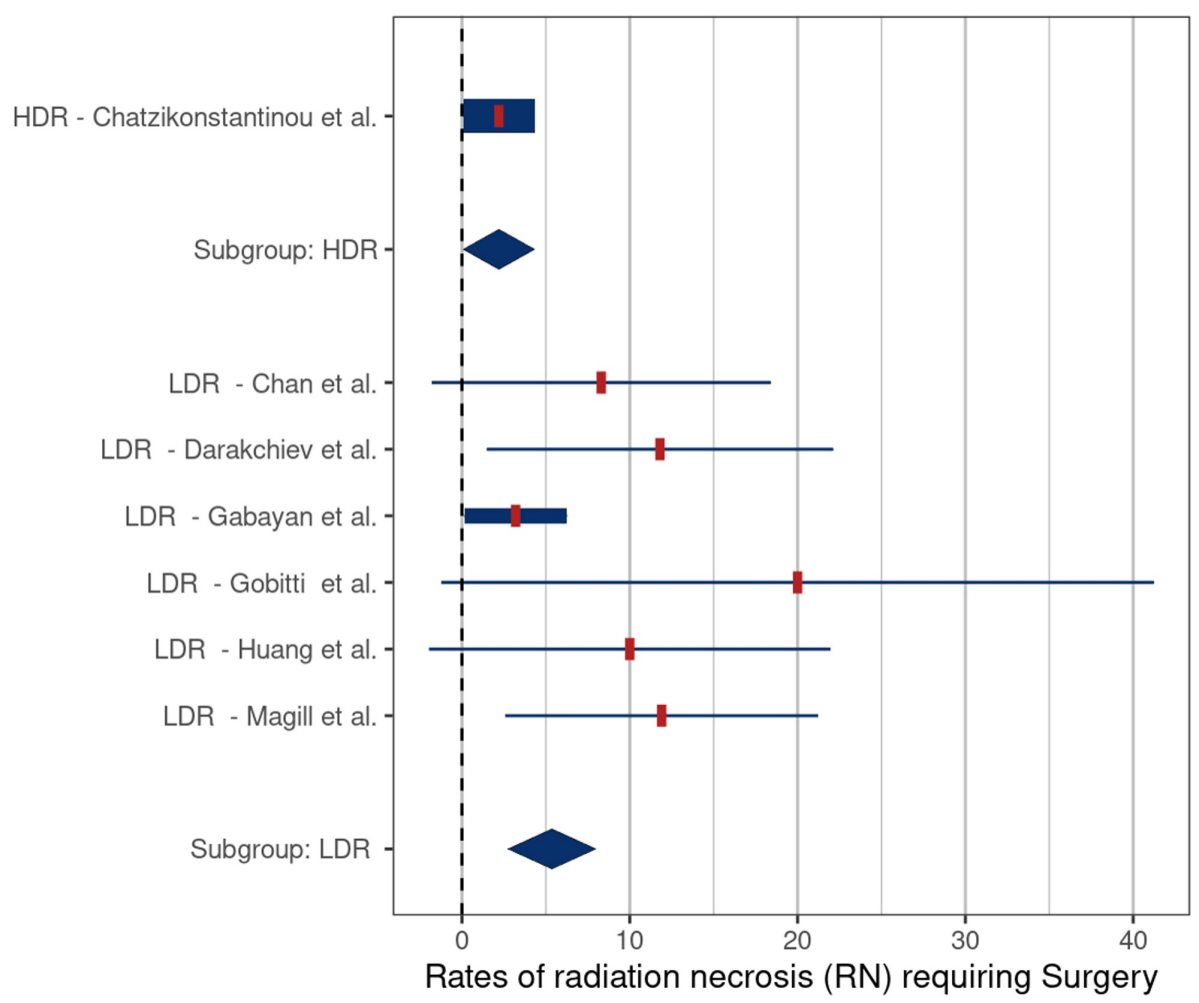
Comparison of rates of radiation necrosis requiring surgery – high-dose-rate (HDR) versus low-dose-rate (LDR) brachytherapy (p = 0.12) Comparison based on included studies [5, 16-19, 21, 27]

The mean rates of symptomatic RN and RN requiring surgery were 2.6% (SE: 1.6) and 5.7% (SE: 1.2) (p = 0.046) (Figure 4) and 2.2% (SE: 2.0) and 5.4% (SE: 1.6) (p = 0.12), respectively, (Figure 5), for HDR versus LDR therapy.

Comparison of tumor types

The rates of symptomatic RN and rates of RN requiring surgery after re-irradiation with brachytherapy were compared for recurrent HGG versus meningioma versus metastases. Detailed descriptions of these populations have already been presented. 

**Figure 6:**
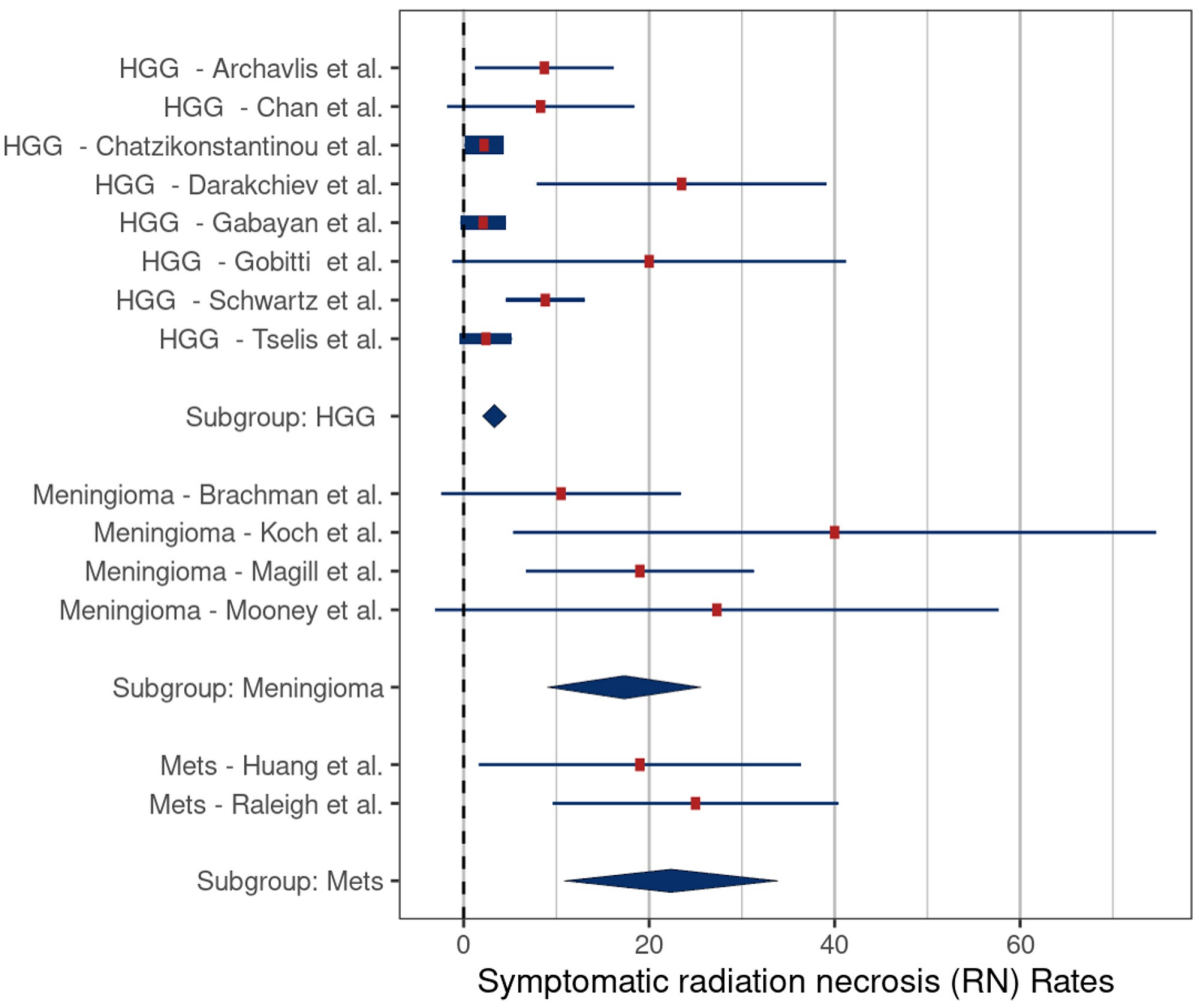
Comparison of rates of symptomatic radiation necrosis – high-grade glioma (HGG) versus meningiomas (p = 0.006) and HGG versus metastases (Mets) (p = 0.007) Comparison based on included studies [3, 5, 16-23, 25-27, 29]

**Figure 7:**
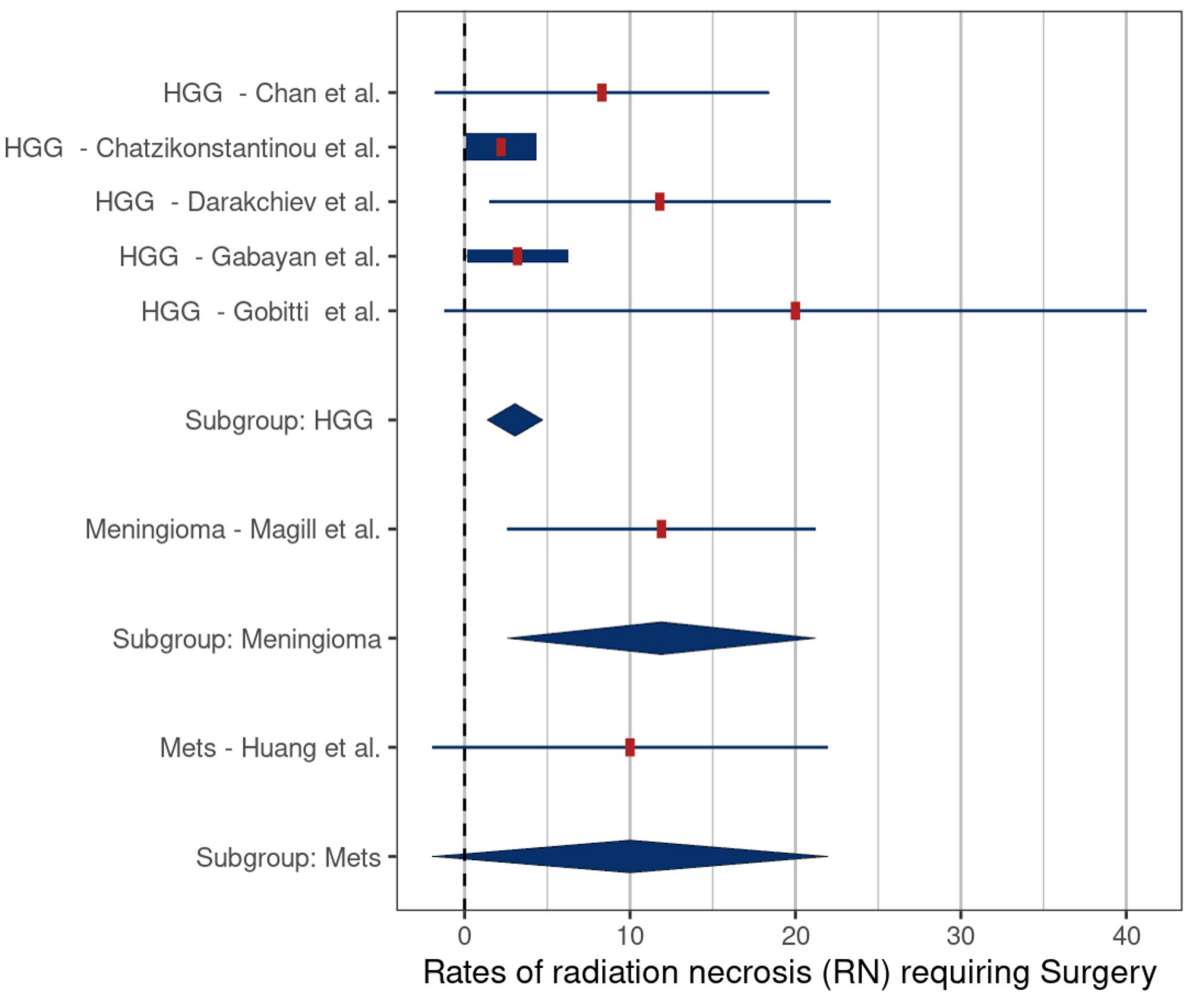
Comparison of rates of radiation necrosis requiring surgery – high-grade gliomas (HGG) versus meningiomas (p = 0.12), and HGG versus metastases (Mets) (p = 0.34) Comparison based on included studies [5, 16-19, 21, 27]

The mean rates of symptomatic RN in the studies treating recurrent HGG, meningiomas, and metastases were 3.3% (SE: 0.8), 17.3% (SE: 5.0), and 22.4% (SE: 7.0), respectively. These rates were significantly different for HGG versus meningiomas (p = 0.006) and for HGG versus metastases (p = 0.007) (Figure 6). The mean rates of RN requiring surgery in the studies treating recurrent HGG, meningiomas, and metastases were 3.0% (SE: 1.0), 11.9% (SE: 5.3), and 10.0% (SE: 7.3), respectively. There were no significant differences in the rates of RN requiring surgery related to tumor types (Figure 7).

## Discussion

The management of locally recurrent brain tumors in previously irradiated patients is a clinical challenge for which no standard of care currently exists [2]. Achieving lasting disease control requires aggressive local therapy which, when feasible, includes re-irradiation [2]. It can be difficult, however, to deliver adequate radiation doses to the target using external beam techniques without causing unacceptable risks of acute and chronic radiation toxicity in the previously treated surrounding brain [4]. Brachytherapy techniques can potentially ameliorate this risk by minimizing the radiation dose to the adjacent tissues while allowing higher doses to be more safely delivered to the tumor and the adjacent brain-tumor interface.

Brachytherapy for recurrent brain tumors can be divided into two primary techniques: interstitial and intracavitary. With interstitial therapy, radiation sources are temporarily or permanently inserted directly into the tumor using stereotactic techniques [9-10]. The most common method involves the transcranial stereotactic placement of catheters in the tumor. The catheters are secured to the scalp and then after loaded with Ir-192/I-125 seeds to deliver a prescription dose of 40 to 50 Gy to the tumor margin [9]. When the source is Ir-192, the prescription dose is given in 5 Gy fractions twice per day, while with I-125, the seeds are left in place continuously for 42 days before explanting the catheters [9].

For intracavitary brachytherapy, the patient undergoes craniotomy with maximum safe resection of the tumor, followed by placement of the radiation source(s) directly into the tumor cavity. With the GliaSite method, a balloon catheter, attached to a subcutaneous reservoir, is left in the tumor resection cavity. After allowing two to six weeks for wound healing, the balloon catheter is filled with a liquid suspension of I-125 and left in place for four to six days to deliver the prescription dose. The liquid I-125 source is then withdrawn, and the balloon catheter system is removed on a delayed basis. This product was withdrawn from the market when it became apparent that the systemic uptake of I-125 from the liquid agent exceeded acceptable safety standards. The remaining reports of intracavitary therapy in this meta-analysis used permanently placed I-125 or Cs-131 seeds. Direct implantation of permanent I-125 or Cs-131 seeds at the time of resection has the benefit of initiating radiation therapy immediately and not requiring an additional procedure for removal. Both I-125 and Cs-131 are gamma emitters with similar intensities (28 versus 30 KeV), but I-125 has a longer half-life than Cs-131 (59.4 days versus 9.7 days, respectively) [11]. It is generally assumed that a radiation source delivers its effective treatment dose over the first five half-lives. For I-125, this is approximately 300 days compared to about 50 days with Cs-131 [11]. The ability to deliver the prescription dose over a much shorter duration gives a theoretical advantage to Cs-131 for rapidly growing tumors [11]. 

We undertook this systematic review and meta-analysis to evaluate the safety and efficacy of the same site reirradiation with modern brachytherapy techniques for recurrent same-site brain tumors. We compared safety outcomes related to RN for interstitial versus intracavity therapy, HDR versus LDR sources, and the three most common recurrent tumors. Analysis of efficacy outcomes was limited by the lack of standardized recording of outcome variables.

Beginning with the interstitial and intracavitary studies, it appears that interstitial therapy is associated with a lower risk of symptomatic RN (3.3% vs 17.7%, respectively) (Table 2); however, the extensive overlap of reported values limited the statistical significance (p = 0.37) (Figure 2). We also found that HDR brachytherapy with Ir-192 had a lower risk of symptomatic RN when compared to treatment with LDR sources (4.4% vs 17%, respectively, p = 0.046). The lower risk of symptomatic RN in these two groups may be related to tumor biology. Among the five interstitial brachytherapy studies, four were performed using HDR (Ir-192) in patients with recurrent HGG. Meta-analysis comparing the rates of symptomatic RN by tumor type demonstrated a significantly lower risk in patients in the recurrent HGG studies compared to those in meningioma studies (3.3% vs. 14.2%, p = 0.006) and HGG versus metastatic tumor studies (3.3% vs. 19.1%, p = 0.007) (Figure 6). One possible reason for the lower rates of symptomatic RN in the HGG patients may be related to the infiltrative nature of these tumors, which makes it difficult to differentiate necrosis from progression and pseudoprogression on routine follow-up imaging studies, leading to underreporting of RN cases. Another possible explanation is that because patients with recurrent glioma have a poor prognosis, they have less time for routine follow-up imaging which, in turn, would lower detection rates for RN. In support of this latter hypothesis, the median OS of patients in the HGG studies was only 9.2 months compared to 26 months in the meningioma studies and 12 months in the metastatic tumor studies (Table 2). 

Despite its potential benefits, brachytherapy is not routinely utilized in the management of recurrent brain tumors. In this systematic review, we identified only 16 published studies that met the inclusion criteria of brachytherapy treatment for recurrent same-site neoplasms in previously radiated patients that met our inclusion criteria (Figure 1). We found that while rates of symptomatic RN as high as 40% were reported for brachytherapy in this population, the pooled mean rate in this analysis was less than 15%. Symptomatic RN rates were lowest for the treatment of recurrent gliomas and in studies that utilized interstitial therapy. While this suggests some advantage for interstitial brachytherapy, the implant technique is challenging and is associated with increased risks of ICH, CSF leak, meningitis, and wound healing complications that have limited its adoption. 

Symptomatic RN rates were highest in recurrent meningioma studies with a mean pooled rate of 24%. An increased risk for re-irradiation is not surprising in this population of Grade II and Grade III meningiomas, many of whom have exhausted their options for external beam treatment. Importantly, several recent studies suggest that brachytherapy significantly improves local control and survival in this group, providing ample support for its continued use [3, 22].

The widespread adoption of brachytherapy for brain tumors has been slow primarily due to the technical demands which, for intracavitary therapy, requires the appropriate spacing and securing of individual seeds (or strands of seeds) to the walls of the tumor resection cavity [7]. Proper spacing of seeds is critical for delivering a safe, effective, and uniform dose of radiation. Recently, a novel brachytherapy device, GammaTile® (GT Medical Technologies, Inc., Tempe, AZ), has become clinically available which minimizes these technical issues [6]. This device consists of Cs-131 seeds positioned 1 cm apart within a collagen carrier tile. The tiles can be rapidly placed after completion of the resection, just prior to closure, typically adding less than five minutes to the case [3].

Study Limitations

The results of this study are limited by the small number of studies available on the same-site re-irradiation using brachytherapy for recurrent brain tumors. We recognized this issue and used a structured meta-analysis approach to minimize the potential biases related to the small sample size and allow evaluation of safety and outcome data. In addition, the time between the first and second RT treatments was not reported in all the selected articles, which complicates the assessment of possible lead-time bias. The confounding issue of lead time bias exists in any evaluation of adverse events that are considered late occurring (such as those from radiation). We believe we have minimized any major impact of underreporting from too short a follow-up by utilizing only studies with a minimum of six months of post-treatment survival. Finally, the authors of this study acknowledge they have potential conflicts of interest. To minimize any bias related to these conflicts, the data analysis was performed by two independent statisticians.

## Conclusions

We have presented a systematic review and meta-analysis of same-site reirradiation with brachytherapy for recurrent brain tumors. Although there is a clear need for additional studies, our analysis of the available literature demonstrates that brachytherapy is safe in well-selected patients. Despite potential benefits, the use of brachytherapy in the management of recurrent brain tumors remains uncommon. The reasons for this may be multifactorial, including the lack of a simple standardized technique and the risks of radiation-related complications with traditional techniques. Recent advances in brain brachytherapy, including the availability of the Cs-131 isotope and the introduction of a new STaRT device that greatly simplifies the placement of seeds, may lead to more widespread adoption. Prospective, randomized trials are needed comparing modern brachytherapy re-irradiation to external beam re-irradiation for recurrent brain tumors. 
